# Functional Model Beverages of Saffron Floral By-Products: Polyphenolic Composition, Inhibition of Digestive Enzymes, and Rheological Characterization

**DOI:** 10.3390/foods13101440

**Published:** 2024-05-07

**Authors:** Débora Cerdá-Bernad, Adrian S. D’costa, Diego A. Moreno, Nicolas Bordenave, María José Frutos

**Affiliations:** 1 Agro-Food Technology Department, CIAGRO-UMH, Centro de Investigación e Innovación Agroalimentaria y Agroambiental, Miguel Hernández University, 03312 Orihuela, Spain; dcerda@umh.es; 2 School of Chemistry and Biomolecular Sciences, Faculty of Sciences, University of Ottawa, Ottawa, ON K1N 6N5, Canada; adcosta@uottawa.ca (A.S.D.); nicolas.bordenave@inrae.fr (N.B.); 3 Phytochemistry and Healthy Food Lab, Department of Food Science and Technology, CEBAS, CSIC, Campus Universitario de Espinardo-25, 30100 Murcia, Spain; dmoreno@cebas.csic.es; 4 School of Nutrition Sciences, Faculty of Health Sciences, University of Ottawa, Ottawa, ON K1H 8L1, Canada; 5 INRAE, Avignon Université, UMR SQPOV, 84000 Avignon, France

**Keywords:** rheology, inhibition assay, phenolic compounds, polysaccharides, aggregation, functional drinks, flavonoids, antioxidants

## Abstract

Despite the rapid and dynamic evolution of research into dietary polyphenols, there is still a knowledge gap regarding their bioaccessibility since it could be influenced by the chemical and nutritional compositions of the food matrix. This study aimed to describe the impact of food thickeners (xanthan gum, guar gum, β-glucan, pectin) on the bioactivity of flavonoids from saffron floral by-products in model beverages before and after thermal processing. The different beverage formulas were characterized in terms of polyphenolic composition using HPLC-DAD-ESI-MS^n^ and rheological properties. The impact of food thickeners and thermal processing on the inhibition of digestive enzymes was also determined. The model beverages mainly presented glycosylated flavonols (of kaempferol, quercetin, and isorhamnetin), with a reduced content in some heat-treated samples. The inhibitory effect on α-amylase was only detected in heat-treated beverages, showing the formulation without any thickener to have the greatest inhibitory effect. Finally, the presence of saffron floral by-products in the beverages showed a tendency to decrease the flow consistency index (*K*) and an increase in the flow behavior index (*n*), most probably driven by the aggregation of phenolics with thickeners. Therefore, this research provides new insights into the development of flavonoid-rich beverages in order to ensure that they exert the expected beneficial effects after their ingestion.

## 1. Introduction

Globally, significant amounts of agricultural by-products, side streams, or harvest remains are treated as low-value material. Efforts to valorize them have become of interest and key to the sustainability of the agri-food system. In particular, they can be turned into high-value-added ingredients for the development of new products, including functional foods [1]. The valorization of saffron (*Crocus sativus* L.) tepals is an archetype of this opportunity since the industrial production of saffron spice only uses the flower’s stigmas. Saffron, in addition to its applications as a spice, has been traditionally used in medicine due to its bioactive content, including carotenoids (crocins), terpenes (safranal), and flavonoids which present beneficial effects due to their potential antioxidant activities [2]. Nevertheless, recent studies have reported that the utilization of saffron industrial by-products may represent an interesting source of bioactive nutrients such as flavonols, flavonoid glycosides, and anthocyanins, and a significant source of income for saffron producers and processors [3]. Therefore, saffron floral bio-residues are valuable natural sources of antioxidant molecules since polyphenols have been positively correlated with the ability to eliminate free radicals and inhibit pro-oxidant enzymes, and recent studies have suggested that the intake of these phytochemicals is associated with the prevention of various diseases, including cancer, cardiovascular, and neurodegenerative diseases [4,5,6,7].

From this perspective, the water-soluble phenolic fraction of saffron tepals could be leveraged into functional beverages able to modulate glycemic response. Indeed, phenolic compounds have been also shown to inhibit digestive amylases, slow down starch digestion, and decrease the rate of intestinal glucose absorption in vitro, thereby providing a viable strategy to manage type 2 diabetes [8,9,10,11].

The development and production of such beverages usually involve formulation with hydrocolloids, such as pectins, xanthan, or guar gum, or even cereal polysaccharides, such as oat β-glucans, in order to adjust their stability and their sensory properties [12]. However, it has been shown that hydrocolloids and phenolic compounds can form non-covalent complexes that affect their respective functionality. Indeed, whereas phenolic compounds are usually partially degraded through thermal processes, it has been shown that complexation with hydrocolloids can protect them from extensive degradation by shielding the phenolics’ peripheral hydroxyl groups from oxidative degradative reactions. Nevertheless, such complexes also sequester phenolic compounds and limit their ability to bind digestive enzymes and glucose transporters, thereby altering their impact on glycemic response. Finally, the formation of hydrocolloid-phenolics non-covalent complexes has also been shown to lead to extensive aggregation of the hydrocolloids, thereby decreasing their ability to build up viscosity and bind water and preventing their adequate use as texture agents and stabilizers [9,13,14].

Nonetheless, all these phenomena seem to be phenolic and hydrocolloid-dependent, and the use of saffron tepal extracts in functional beverages requires elucidating their behavior in realistic beverage models incorporating hydrocolloids as texture agents. With this in mind, the current study describes the impact of food thickeners on the bioactivity of flavonoids from saffron floral by-products and their ability to modulate starch digestion before and after thermal processing (TP) (10 min, 80 °C). The specific objectives were settled as follows: (1) characterization of the phenolic composition of saffron floral by-products and saffron floral by-products-model beverages by HPLC-DAD-ESI-MS^n^; (2) determination of the impact of food thickeners and thermal processing on the inhibition of the digestive enzyme (α-amylase); and (3) characterization of the rheological properties of saffron floral-model beverages which could influence the bioaccessibility of polyphenols, and to provide in-depth information on the structural changes associated with processing of foods upon thermal treatment. A detailed understanding of such food matrix factors was explored to evaluate the positive health-promoting effects of these antioxidant functional beverages.

## 2. Materials and Methods

### 2.1. Chemicals and Reagents

The polysaccharides β-glucan (P-BGBM), guar gum (P-GGMMV), and xanthan gum (P-XANTH) were purchased from Cedarlane (Burlington, ON, Canada). Pectin from apples (poly-D-galacturonic acid methyl ester) was purchased from Sigma-Aldrich (St. Louis, MO, USA). D-(+)-Sucrose (purity 99%) was obtained from TCI America (Portland, OR, USA) and citric acid monohydrate from Fisher Chemical (Hampton, NH, USA). The enzyme ⍺-amylase from porcine pancreas (PPA, type VI-B, 15 units/mg solid), amylopectin from maize (purity > 99%), maltose monohydrate, disodium hydrogen phosphate, sodium dihydrogen phosphate, sodium chloride, potassium sodium tartrate, 3,5-dinitrosalicylic acid, and sodium hydroxide were from Millipore-Sigma (Oakville, ON, Canada). Standard compounds, methanol, acetonitrile, and formic acid HPLC grade were purchased from Sigma-Aldrich (St. Louis, MO, USA). All chemicals used were of analytical grade, and all solutions were prepared with MilliQ water.

### 2.2. Plant Material

Saffron floral by-products were obtained from the Castilla-La Mancha region (Spain) and were composed of tepals, stamens, and styles, with the stigmas being detached manually. Saffron floral by-products were freeze-dried for 48 h, crushed, sieved (500 μm mesh size), and kept at −20 °C until further analysis.

### 2.3. Preparation of Model Beverages

The model beverages were prepared using potable water (>90% *w*/*w*), sucrose (7% *w*/*w*), citric acid (0.20% *w*/*w*), food thickener (0.25% *w*/*w*) (xanthan gum, guar gum, β-glucan, pectin; no thickener as control for the effect of thickeners), and freeze-dried saffron floral by-products (1% *w*/*w*). For each thickener, a model beverage was prepared, and a control sample including all the ingredients except saffron flowers was also elaborated. The model beverages were prepared first by mixing food thickeners with water under stirring, following dissolution instructions from suppliers. Then, the rest of the ingredients were added under stirring until completely dissolved. Samples were centrifugated (11,200× *g*, 20 min) (Eppendorf Centrifuge 5804/5804R, Sigma Aldrich, St. Louis, MO, USA) to remove insoluble compounds. Furthermore, to study the effect before and after thermal processing, some of the samples were thermally treated at 80 °C for 10 min in a water bath (Unitronic 200, J.P. Selecta, Barcelona, Spain) and cooled to 37 °C in an ice bath. 

A total of 20 different beverages, including control samples, were obtained:

- Control (water, sucrose, citric acid) without thickener non-heat-treated (C), model beverage without thickener non-heat treated (SF), control without thickener heat-treated (C80), model beverage without thickener heat-treated (SF80).
- Control (water, sucrose, citric acid) with xanthan gum non-heat-treated (XGC), model beverage with xanthan gum non-heat treated (XGSF), control with xanthan gum heat-treated (XGC80), model beverage with xanthan gum heat-treated (XGSF80).
- Control (water, sucrose, citric acid) with guar gum non-heat-treated (GGC), model beverage with guar gum non-heat treated (GGSF), control with guar gum heat-treated (GGC80), model beverage with guar gum heat-treated (GGSF80).
- Control (water, sucrose, citric acid) with β-glucan non-heat-treated (BGC), model beverage with β-glucan non-heat treated (BGSF), control with β-glucan heat-treated (BGC80), model beverage with β-glucan heat-treated (BGSF80).
- Control (water, sucrose, citric acid) with pectin non-heat-treated (PC), model beverage with pectin non-heat treated (PSF), control with pectin heat-treated (PC80), model beverage with pectin heat-treated (PSF80).
- Each beverage formulation was made in triplicate.

### 2.4. Preparation of Extracts for Phenolics Identification and Quantification

Freeze-dried saffron floral by-products were prepared for HPLC analysis, and 100 mg of dried material were weighed for extraction with 1 mL of 50% methanol acidified with 1% formic acid, mixed 30 s vortex, sonicated for 1 h (Branson 551021 Sigma-Aldrich, St. Louis, MO, USA), and subjected to a 24 h overnight at 4 °C. Then, samples were centrifuged at 10,000 rpm for 15 min (Hettich^®^ EBA 21 Sigma-Aldrich, St. Louis, MO, USA). Supernatants were collected and filtered through a 0.22 μmØ polytetrafluoroethylene membrane (Millipore, Bedford, MA, USA) and stored at −20 °C until further analysis. 

Beverage samples were directly used for HPLC analysis, having been previously filtered through a 0.22 μmØ polytetrafluoroethylene membrane (Millipore, Bedford, MA, USA) and stored at −20 °C until further analysis. 

### 2.5. Identification and Quantification of Phenolic Compounds by HPLC-DAD-ESI/MS^n^

Identification of the phenolic compounds was performed with an Agilent HPLC 1200 series model equipped with a photodiode array detector (model G1315B), a mass detector in series (Agilent Technologies, Waldbronn, Germany), a binary pump (model G1312A), a degasser (model G1322A) and an autosampler (model G1313A), based on a method described Gonçalves, Campos, Alves, Garcia-Viguera, Moreno and Silva [15]. The mass detector was an ion trap spectrometer (model G2445A) equipped with an electrospray ionization interface and was controlled by LC/MS software (Esquire Control Ver. 6.1. Build No. 534.1., Bruker Daltoniks GmbH, Bremen, Germany). A Nucleosil^®^ 100–5 C18 column (25.0 cm × 0.46 cm; 5 μm particle size waters; Macherey-Nagel, Düren, Germany) was used. The mobile phase, pumped at a flow rate of 0.8 mL/min, consisted of 1% aqueous formic acid (solvent A) and acetonitrile (solvent B). The solvent system started with 8% of B and reached 15% of B at 25 min, 22% at 55 min, and 40% at 60 min, with a washout period of 5 min, and it returned to initial conditions afterward. Mass spectra were acquired with a scan range from m/z 100 to 1200, and MS parameters were set as follows: the capillary temperature was 350 °C, capillary voltage was set at 4 kV, nebulizer pressure was 65.0 psi, and the nitrogen flow rate was 11 L/min. Collision-induced fragmentation experiments were performed in an ion trap using helium as collision gas, with voltage ramping cycles from 0.3 to 2 V. For anthocyanins, mass spectrometry data were acquired in positive ionization mode while for non-colored phenolics, acquisition was performed in a negative ionization mode. MS^n^ was carried out in automatic mode on more abundant fragment ions in MS^(n−1)^. HPLC system was controlled by ChemStation for LC 3D Systems software Rev. B.01.03-SR2 (204) (Agilent Technologies Spain S.L., Madrid, Spain). Injections (20 μL) of each sample were performed in triplicate.

Spectral data from all peaks were accumulated in a range of 200–600 nm and chromatograms were recorded at 360 nm (flavonols) and 520 nm (anthocyanins), and compounds in each sample were tentatively identified based on their elution order retention times, and ultraviolet-visible and mass spectra features as compared to authentic standards analyzed under same conditions and data available in the literature. Identified compounds were finally quantified using calibration curves of the standard reference compounds, when available, or using the most structurally related reference compound, built in a concentration range of 0.06 to 1 mmol/L.

### 2.6. ⍺-amylase In-vitro Inhibition Assay

The inhibition assay was carried out according to D’Costa and Bordenave [16] with minor modifications, using 50 μL of PBS buffer (blank) or 50 μL of each sample. The model beverages were previously neutralized, according to Freitas and Le Feunteun [8], because the pH of the chime is quickly neutralized in the duodenum, so it is unlikely that pancreatic enzymes are affected by the native pH of foods. The amount of maltose generated by this assay in the control beverage without thickener and without heat treatment (C) was used as a reference corresponding to 100% PPA activity. In other beverage systems, the amount of maltose released through the assay was expressed as a percentage of this reference PPA activity based on the amount of maltose released from the control beverage.

After the reaction, samples (250 μL) were transferred into a 96 well micro-plate and the absorbance was read at 540 nm at 25 °C (Tecan Spark multimode micro-plate reader, Baldwin Park, CA, USA). Absorbance reading was converted into a maltose concentration with a maltose standard curve (R^2^ = 0.9982), with maltose concentration ranging from 0 to 1 mg/mL.

### 2.7. Rheology Measurements

Rheological characterization of beverages was performed using a Discovery series Hybrid Rheometer (HR-2) from TA Instruments (New Castle, DE, USA) to measure concentric cylinder geometry. For each test solution, shear stress (τ in Pa) vs. shear rate (γ in s^−1^) profiles were measured for shear rate ranging from 10^−1^ to 10^3^ s^−1^. 

These profiles were fitted to a Herschel–Bulkley power law, τ = τ_0_ + Kγ^n^ by the least-square method using the Solver function on Microsoft Excel 365 (Microsoft, Redmond, WA, USA). The flow consistency index (*K*, in Pa·s^−n^) and flow behavior index (*n*, dimensionless) of the solutions were extracted from this non-linear regression method.

### 2.8. Statistical Analysis

All experiments were carried out in triplicate. Results were expressed as mean ± standard deviation. The mean comparisons were performed using two-way analysis of variance (ANOVA) and Tukey’s multiple range test, using SPSS version 21.0 software package (SPSS Inc., Chicago, IL, USA). The significant differences were established as *p* ≤ 0.05.

## 3. Results and Discussion

### 3.1. Phenolic Profile of Saffron Floral By-Products and Model Beverages

Saffron floral by-products and the model beverages prepared without adding food thickeners (SF, SF80) were first characterized for their profiles in native polyphenols. As reported in Table 1 and Table 2, a total of 14 and 13 polyphenolic compounds were identified or tentatively identified and quantified in saffron floral by-products and in the model beverages, respectively. Regarding the flavonol fraction in saffron floral extracts (Table 1), it was composed by kaempferol, quercetin and isorhamnetin glycosides. Kaempferol-*O*-sophoroside resulted in the most abundant polyphenolic compound (1058 ± 38 mg/100 g), followed by quercetin-3,4′-*O*-diglucoside (II) (222 ± 12 mg/100 g) and kaempferol-3-*O*-sophoroside-7-*O*-glucoside (204 ± 16 mg/100 g).

Glycosides of isorhamnetin, like isorhamnetin-3,7-di*O*-hexoside (I), isorhamnetin-3,7-di*O*-hexoside (II), and isorhamnetin-3-*O*-rutinoside, were also present but in lower concentrations (42–74 mg/100 g). Moreover, four anthocyanins were identified and quantified in the extracts of saffron floral by-products, including petunidin and delphinidin derivatives. Delphinidin-3,5*O*-diglucoside, followed by delphinidin-3-*O*-glucoside, were the main anthocyanins found in saffron floral extracts, showing a concentration of 145 ± 13 and 80.7 ± 6.8 mg/100 g, respectively. Petunidin-3,5-*O*-diglucoside and petunidin-3-*O*-glucoside were present in lower amounts (43.5 ± 3.7 and 23.9 ± 1.4 mg/100 g, respectively). These results were in accordance with previous studies that reported the highest content for kaempferol-*O*-sophoroside and delphinidin-di-*O*-glucoside in saffron floral bio-residues, with the flavonol fraction being mainly composed of kaempferol derivatives [17,18,19].

Concerning saffron floral model beverages, the same composition of polyphenols as saffron floral extracts was found, except quercetin-3-O-glucoside, but with lower concentrations of each compound, since beverages contained 1% of saffron floral by-products (Table 2). Nevertheless, statistically significant differences were observed in the concentration of some compounds between the heat-treated (SF80) and the non-heat-treated (SF) model beverages. Both model beverages were mainly composed of kaempferol, quercetin, and isorhamnetin glycosides, with kaempferol-3-*O*-sophoroside being the major one found, showing SF statistically significant higher amounts (22.9 ± 0.6 mg/100 mL) compared to the heat-treated beverage SF80 (21.6 ± 0.4 mg/100 mL). Kaempferol-3-*O*-sophoroside-7-*O*-glucoside was also present in significantly higher concentrations in the model beverage non-heat-treated (5.57 ± 0.11 mg/100 mL) with respect to the heat-treated sample (5.27 ± 0.06 mg/100 mL). In addition to kaempferol sophoroside-derivatives, quercetin-3,4′-*O*-diglucoside (II) was detected in elevated amounts (4.1–4.6 mg/100 mL). Regarding anthocyanins composition, petunidin and delphinidin derivatives were also present in saffron floral model beverages, with delphinidin-3,5-*O*-diglucoside and delphinidin-3-*O*-glucoside being the major ones. 

The model beverage non-heat-treated showed statistically significant higher amounts of delphinidin-3-*O*-glucoside, petunidin-3,5-*O*-diglucoside and petunidin-3-*O*-glucoside compared to the heat-treated beverage. These significant differences found in the polyphenol content of saffron floral by-products model beverages could be related to the degradation of polyphenols upon thermal processing and to the acidic environment of the system. Lower pH may cause the degradation of flavonoid glycosides under thermal conditions [13,14,20]. These results exhibited trends similar to those of previous research in which the thermal processing significantly affected individual polyphenolic compounds [21].

Therefore, these data showed that *C. sativus* floral by-products are a valuable source of potentially interesting phytochemicals like flavonoids and could constitute a workable source of antioxidant compounds to develop antioxidant-functional beverages. Flavonoids have been found to be strong antioxidants that can neutralize free radicals by donating an electron or a hydrogen atom. However, their bioactivity and beneficial health effects depend on their bioaccessibility, which needs to be studied considering their interaction with polysaccharides. It is important to obtain information about their bioaccessibility from the foods matrix and the factors that could influence it in order to ensure that these antioxidant-functional beverages exert positive effects on the health of the host. 

### 3.2. Inhibitory Effect of Saffron Floral by-Products Model Beverages on the Enzymatic Activity of Pancreatic α-Amylase

**Figure 1:**
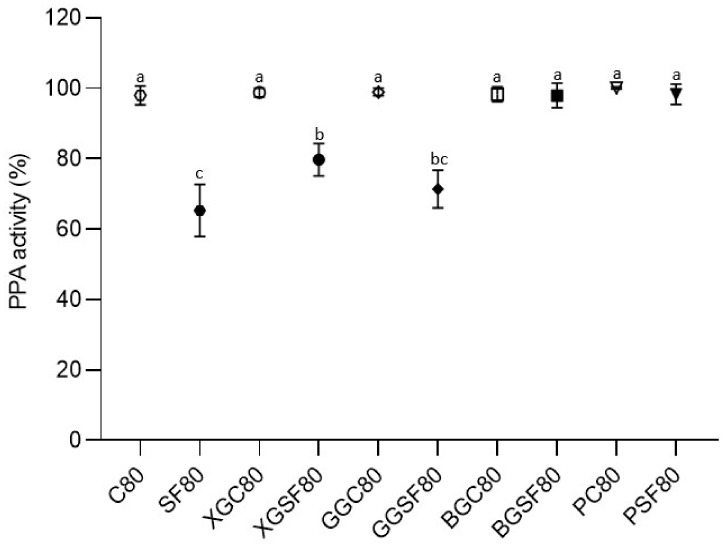
Impact of saffron floral by-products model beverages on the activity of pancreatin α-amylase. Data represent means ± standard deviation and different lowercase letters indicate statistically significant differences at *p* ≤ 0.05 for each model beverage; C80: control without thickener heat-treated; SF80: model beverage without thickener heat-treated; XGC80: control with xanthan gum heat-treated; XGSF80: model beverage with xanthan gum heat-treated; GGC80: control with guar gum heat-treated; GGSF80: model beverage with guar gum heat-treated; BGC80: control with β-glucan heat-treated; BGSF80: model beverage with β-glucan heat-treated; PC80: control with pectin heat-treated; PSF80: model beverage with pectin heat-treated.

Due to the polyphenolic composition of saffron floral by-products model beverages, their capacity to inhibit the activity of digestive amylases, such as pancreatic α-amylase, was studied. Functional foods rich in polyphenols could be potential candidates for improving glucose homeostasis by reducing intestinal absorption of dietary glucose through the inhibition of digestive enzymes, thereby slowing down starch digestion [11]. The inhibitory effect of the tested saffron floral by-products model beverages on the activity of PPA is presented in Figure 1 as a percentage of PPA activity in the control beverage.

In the non-heat-treated samples, no inhibitory effect was detected in any model beverage, thus only the results of samples after thermal processing are shown in Figure 1.

No PPA inhibitory activity was detected in heat-treated control beverages without saffron floral by-products, as expected. No statistically significant PPA inhibitory activity was detected either in the BGSF80 or the PSF80 model. The heat-treated model beverage without thickener SF80 showed the greatest inhibitory effect on PPA (~37%) since PPA activity was 62.46 ± 7.18%, followed by GGSF80 and XGSF80. The heat-treated model beverage with guar gum also presented similar inhibitory capacity on PPA (~29%), with the PPA activity being 71.42 ± 5.38%, without a statistically significant difference compared to SF80. However, statistically significant differences were found between XGSF80 and SF80, presenting inhibitory levels of about 20% on PPA, as the α-amylase activity in XGSF80 was 79.75 ± 4.59%. 

All model beverages contained 1% of phenolic-rich saffron floral by-products, did not differ in their sugar composition, and although the model beverages differed in their pH, the PPA assays were all conducted after pH neutralization, reflecting in vivo pH neutralization of the chime once it is propelled into the duodenum [8]. Therefore, differences in PPA inhibition could only be due to the addition of food thickener and the thermal processing. 

Polyphenols can inhibit α-amylase through binding with it. However, the presence of polysaccharides, such as those naturally present in saffron flowers and the added food thickeners, could introduce competitive binding opportunities for the phenolic compounds and decrease polyphenol-enzyme binding, and consequently decrease the ability of polyphenols to inhibit the enzyme [22,23]. This has been shown in previous studies with soluble fibers such as β-glucan, pectins, arabinoxylans, starch itself, as well as xanthan and guar gum [16,23,24,25]. Our results are consistent with these findings with the observation that the model beverage without thickener (therefore without polysaccharide to interfere with enzyme-polyphenol binding and inhibition) showed the greatest PPA inhibition. 

However, temperature also played an important role, as it appears that all samples that were heat-treated did not exhibit PPA inhibition. This observation suggests that in heat-treated samples, polyphenols were available enough to bind (and inhibit) PPA as if polysaccharides were not present, although they were. The thermal processing could have affected the hydrogen-bonding driving polysaccharides-polyphenols interactions, and molecular thermal agitation may prevent polysaccharide-polyphenol binding, which is known to be weaker than polyphenol-protein binding [26]. This could explain why heat-treated model beverages, such as SF80, XGSF80, and GGSF80, showed an inhibitory effect on digestive enzymes. Therefore, polyphenol-polysaccharide complexation had a direct effect on the inhibition of digestive enzyme activity by phenolic compounds, but further research is needed in order to unveil the influence of other factors and how they impact the functional properties of food products, altering the functionality of polyphenols.

### 3.3. Rheological Properties of Saffron Floral by-Products Model Beverages 

Rheological properties play an important role in developing new functional food products by providing valuable information on the structural changes associated with food processing and allowing us to understand the interaction of food components [27]. 

Rheological properties of the saffron floral by-products model beverages were characterized from the fitting of stress vs. shear rheograms fitted with a Herschel–Bulkley power law, and the results are reported in Table 3. 

In these results, the flow behavior index, *n*, characterizes the behavior of a solution under shear (shear-thinning for *n* < 1, Newtonian for *n* = 1, shear-thickening for *n* > 1). The flow consistency index, *K*, is reflective of the viscosity of a solution at a comparable shear rate and flow behavior index.

Results reported in Table 3 for solutions without saffron extract show that control solutions were essentially Newtonian, as were beverages with β-glucans and pectins (*n* ≈ 1), which also exhibited similarly low viscosity (similar consistency index K). Conversely, model beverages containing xanthan and guar gums exhibited marked shear-thinning behavior (*n* ≈ 0.4–0.9) and marked viscosity. The addition of saffron floral by-products rich in polyphenols to model beverages thickened with guar and xanthan gums led to a decrease in flow consistency index *K* and an increase in flow behavior index *n*. This has been observed before and has been attributed to phenolic-driven aggregation of thickeners, leading the solution to behave more like water in terms of flow and viscosity [28,29]. Specifically, in model beverages with xanthan gum, thermal processing (XGC80, XGSF80) tends to significantly decrease *K* and τ_0_, which is reflective of the minimum stress needed to apply to the liquid so it starts flowing. Thermal processing and saffron floral by-products (XGSF80) tend to significantly increase *n*. In model beverages thickened with guar gum, saffron floral by-products (GGSF, GGSF80) tend to significantly increase *τ*_0_ and *n*, and, combined with thermal processing, tend to significantly decrease *K* (GGSF, GGC80, GGSF80). The results concerning model beverages with β-glucans were consistent with those of guar and xanthan gum, but they had very limited sensitivity to the presence of saffron floral by-products. This fact may be related to the fact that β-glucans bind less with flavonols, the major polyphenols present in the studied beverages [30]. 

As expected, model beverages with pectin showed the opposite behavior to xanthan gum, guar gum, and β-glucans since pectin was the only strongly ionic gum in this study, which may impact the nature of interactions with phenolic compounds. Saffron floral by-products (PSF) tend to significantly increase *K* and *τ*_0_ and decrease *n*. However, for model beverages without thickener, no statistically significant variations were found for *K* and *n*, regarding the presence of saffron floral by-products and the thermal processing. 

It must be noted that these observations about the effect of thermal processing-saffron floral by-products interactions could be confounded by paradoxical effects, with possible heat degradation of saffron phenolic compounds on the one hand (decreasing their effect of the rheological properties of the beverages) and potentially enhanced hydration/dispersion of food thickeners on the other hand (increasing potential binding with polyphenols). Nevertheless, further detailed examination is needed to bring additional insights into the binding and aggregation mechanisms. From the point of view of the impact of polyphenol-polysaccharide interactions on these model beverages, the reported observations are important as they may influence food processing and formulation choices depending on food thickener composition requirement and phenolic content of the formulation. Indeed, in these saffron floral by-products model beverages, aggregation may lead to drastically different effects depending on the type of food thickener chosen. 

## 4. Conclusions

A detailed understanding of how a complex food matrix, like saffron floral model beverages, influences the rheological properties and content of polyphenols was explored, providing new information. The studied model beverages mainly presented glycosylated flavonols of kaempferol, quercetin, and isorhamnetin, and the inhibitory effect on α-amylase was only detected in heat-treated beverages. Furthermore, the presence of saffron floral by-products showed a decreasing tendency in the flow consistency index (*K*) and an increase in the flow behavior index (*n*). These changes in the rheological properties were most probably driven by the aggregation of phenolics with thickeners, demonstrating that the addition of food thickeners in beverage formulations plays a key role due to the polysaccharide-polyphenol interactions. These conclusions may be important to the development of new antioxidant food products rich in phenolic compounds where gums or fibers are used as thickening agents in order to ensure that they exert the expected beneficial effects after their ingestion. Additionally, the possibility of using dried saffron floral by-products without any additional processing could be an important issue for the food industry, contributing to minimizing the environmental impact.

## Figures and Tables

**Table 1:** Chromatographic, mass spectra characteristics and quantification of native polyphenols identified in saffron floral by-product extracts. Data are expressed as mean ± SD (*n* = 3).

Compound	RT (min)	[M]^+^ (m/z)	[M − H]^−^ (m/z)	MS^n^ (m/z)	Concentration (mg/100 g dw)
**Flavonols**					
Kaempferol-3-*O*-sophoroside-7-*O*-glucoside	7.50		771	609, 285, 429	204 ± 16
Isorhamnetin-3,7-diO-hexoside (I)	13.63		639/641	315, 271	44.9 ± 4.5
Quercetin-3,4′-*O*-diglucoside (I)	16.03		625	463, 301	70.6 ± 2.7
Quercetin-3,4′-*O*-diglucoside (II)	16.45		625	301, 463, 445	222 ± 12
Isorhamnetin-3,7-diO-hexoside (II)	17.30		639	315, 477, 300	74.3 ± 2.7
Kaempferol-3-*O*-sophoroside	18.95		609	285, 429	1058 ± 38
Quercetin-3-*O*-glucoside	21.51		463	301, 151	42.6 ± 4.2
Isorhamnetin-3-*O*-rutinoside	21.68		623	315, 459	71.5 ± 4.2
Kaempferol-3-*O*-(acetyl-glycoside)-7-*O*-glycoside	24.66		651	285, 489, 471	103 ± 5
Kaempferol-3-*O*-glucoside	24.88		447	285	151 ± 8
**Anthocyanins**					
Delphinidin-3,5-*O*-diglucoside	5.05	627		465, 303	145 ± 13
Petunidin-3,5-*O*-diglucoside	7.21	641		479, 317	43.5 ± 3.7
Delphinidin-3O-glucoside	8.96	465		303	80.7 ± 6.8
Petunidin-3-*O*-glucoside	12.02	479		317	23.9 ± 1.4

RT: retention time; m/z: mass to charge.

**Table 2:** Chromatographic, mass spectra characteristics and quantification of native polyphenols identified in saffron floral by-product model beverages without thickener.

Compound	RT (min)	[M]^+^ (m/z)	[M − H]^−^ (m/z)	MS^n^ (m/z)	Concentration (mg/100 mL of Beverage)	
**Flavonols**					SF	SF80
Kaempferol-3-*O*-sophoroside-7-*O*-glucoside	7.50		771	609, 285, 429	5.57 ± 0.11 ^a^	5.27 ± 0.06 ^b^
Isorhamnetin-3,7-diO-hexoside (I)	13.63		639/641	315, 271	2.71 ± 0.12	2.74 ± 0.01
Quercetin-3,4′-*O*-diglucoside (I)	16.03		625	463, 301	2.85 ± 0.02	2.82 ± 0.02
Quercetin-3,4′-*O*-diglucoside (II)	16.45		625	301, 463, 445	4.64 ± 0.10 ^a^	4.17 ± 0.09 ^b^
Isorhamnetin-3,7-diO-hexoside (II)	17.30		639	315, 477, 300	2.93 ± 0.04 ^a^	2.85 ± 0.02 ^b^
Kaempferol-3-*O*-sophoroside	18.95		609	285, 429	22.9 ± 0.6 ^a^	21.6 ± 0.4 ^b^
Isorhamnetin-3-*O*-rutinoside	21.68		623	315, 459	2.85 ± 0.01 ^a^	2.81 ± 0.02 ^b^
Kaempferol-3-*O*-(acetyl-glycoside)-7-*O*-glycoside	24.66		651	285, 489, 471	3.08 ± 0.01 ^a^	3.02 ± 0.02 ^b^
Kaempferol-3-*O*-glucoside	24.88		447	285	3.37 ± 0.03	3.33 ± 0.04
**Anthocyanins**						
Delphinidin-3,5-*O*-diglucoside	5.05	627		465, 303	3.57 ± 0.37	3.44 ± 0.07
Petunidin-3,5-*O*-diglucoside	7.21	641		479, 317	1.29 ± 0.03 ^a^	1.23 ± 0.02 ^b^
Delphinidin-3O-glucoside	8.96	465		303	1.67 ± 0.03 ^a^	1.60 ± 0.02 ^b^
Petunidin-3-*O*-glucoside	12.02	479		317	0.88 ± 0.00 ^a^	0.86 ± 0.00 ^b^

Means ± standard deviation in the same row followed by different lowercase letters indicate statistically significant differences at *p* ≤ 0.05 for each model beverage; RT: retention time; m/z: mass to charge; SF: model beverage without thickener non-heat treated; SF80: model beverage without thickener heat-treated.

**Table 3:** Flow parameters of saffron floral by-products model beverages fitted using Herschel and Bulkley.

	*τ*_0_ (Pa)	*K* (Pa·s^n^)	*n*
C	0.56 ± 0.62 ^a^	0.19 ± 0.03	1.01 ± 0.01
SF	1.4 ± 1.6 ^ab^	0.18 ± 0.02	1.02 ± 0.01
C80	0.78 ± 0.68 ^ab^	0.20 ± 0.05	1.01 ± 0.02
SF80	0.0 ± 0.0 ^b^	0.22 ± 0.04	1.00 ± 0.02
XGC	750 ± 70 ^a^	294 ± 7 ^b^	0.39 ± 0.00 ^bc^
XGSF	615 ± 71 ^b^	342 ± 33 ^a^	0.38 ± 0.01 ^c^
XGC80	115 ± 4 ^c^	167 ± 45 ^c^	0.42 ± 0.02 ^b^
XGSF80	206 ± 11 ^c^	1.52 ± 0.11 ^d^	0.84 ± 0.01 ^a^
GGC	0.0 ± 0.0 ^c^	95.3 ± 5.7 ^a^	0.48 ± 0.00 ^c^
GGSF	121 ± 4 ^a^	1.54 ± 0.29 ^c^	0.87 ± 0.02 ^a^
GGC80	0.0 ± 0.0 ^c^	77.2 ± 4.0 ^b^	0.50 ± 0.00 ^c^
GGSF80	90.9 ± 2.6 ^b^	9.42 ± 0.80 ^c^	0.70 ± 0.01 ^b^
BGC	38.0 ± 1.8 ^a^	0.14 ± 0.02	1.09 ± 0.01 ^a^
BGSF	12.8 ± 3.5 ^b^	0.17 ± 0.02	1.05 ± 0.01 ^b^
BGC80	39.3 ± 5.4 ^a^	0.14 ± 0.02	1.09 ± 0.02 ^a^
BGSF80	33.4 ± 1.7 ^a^	0.17 ± 0.01	1.07 ± 0.01 ^ab^
PC	8.2 ± 1.3 ^a^	0.19 ± 0.02 ^b^	1.03 ± 0.01 ^a^
PSF	287 ± 256 ^b^	1.06 ± 0.12 ^a^	0.88 ± 0.01 ^b^
PC80	10.0 ± 0.9 ^a^	0.18 ± 0.01 ^b^	1.03 ± 0.01 ^a^
PSF80	8.3 ± 7.3 ^a^	0.20 ± 0.02 ^b^	1.02 ± 0.02 ^a^

Means ± standard deviation in the same row followed by different lowercase letters indicate statistically significant differences at *p* ≤ 0.05 for each model beverage; C: control without thickener non-heat-treated; SF: model beverage without thickener non-heat treated; C80: control without thickener heat-treated; SF80: model beverage without thickener heat-treated; XGC: control with xanthan gum non-heat-treated; XGSF: model beverage with xanthan gum non-heat treated; XGC80: control with xanthan gum heat-treated; XGSF80: model beverage with xanthan gum heat-treated; GGC: control with guar gum non-heat-treated; GGSF: model beverage with guar gum non-heat treated; GGC80: control with guar gum heat-treated; GGSF80: model beverage with guar gum heat-treated; BGC: control with β-glucan non-heat-treated; BGSF: model beverage with β-glucan non-heat treated; BGC80: control with β-glucan heat-treated; BGSF80: model beverage with β-glucan heat-treated; PC: control with pectin non-heat-treated; PSF: model beverage with pectin non-heat treated; PC80: control with pectin heat-treated; PSF80: model beverage with pectin heat-treated.

## Data Availability

The original contributions presented in the study are included in the article, further inquiries can be directed to the corresponding author.
